# Eprobe Mediated Real-Time PCR Monitoring and Melting Curve Analysis

**DOI:** 10.1371/journal.pone.0070942

**Published:** 2013-08-07

**Authors:** Takeshi Hanami, Diane Delobel, Hajime Kanamori, Yuki Tanaka, Yasumasa Kimura, Ayako Nakasone, Takahiro Soma, Yoshihide Hayashizaki, Kengo Usui, Matthias Harbers

**Affiliations:** 1 Division of Genomic Technologies, RIKEN Center for Life Science Technologies, Yokohama, Kanagawa, Japan; 2 K.K. DNAFORM, Yokohama, Kanagawa, Japan; 3 RIKEN Preventive Medicine and Diagnosis Innovation Program, Yokohama, Kanagawa, Japan; Naval Research Laboratory, United States of America

## Abstract

Real-time monitoring of PCR is one of the most important methods for DNA and RNA detection widely used in research and medical diagnostics. Here we describe a new approach for combined real-time PCR monitoring and melting curve analysis using a 3′ end-blocked Exciton-Controlled Hybridization-sensitive fluorescent Oligonucleotide (ECHO) called Eprobe. Eprobes contain two dye moieties attached to the same nucleotide and their fluorescent signal is strongly suppressed as single-stranded oligonucleotides by an excitonic interaction between the dyes. Upon hybridization to a complementary DNA strand, the dyes are separated and intercalate into the double-strand leading to strong fluorescence signals. Intercalation of dyes can further stabilize the DNA/DNA hybrid and increase the melting temperature compared to standard DNA oligonucleotides. Eprobes allow for specific real-time monitoring of amplification reactions by hybridizing to the amplicon in a sequence-dependent manner. Similarly, Eprobes allow for analysis of reaction products by melting curve analysis. The function of different Eprobes was studied using the L858R mutation in the human epidermal growth factor receptor (EGFR) gene, and multiplex detection was demonstrated for the human EGFR and KRAS genes using Eprobes with two different dyes. Combining amplification and melting curve analysis in a single-tube reaction provides powerful means for new mutation detection assays. Functioning as “sequence-specific dyes”, Eprobes hold great promises for future applications not only in PCR but also as hybridization probes in other applications.

## Introduction

Many PCR applications directly monitor the amplification reaction in real-time such as research applications, commercial tests, and medical diagnostics. This can be achieved by fluorescent dyes like SYBR Green I [1] that intercalate into the synthesized double-stranded DNA. Such dyes are very sensitive, but they lack any sequence specificity and their signal is not amplicon dependent. Therefore primer dimers and other PCR artifacts can lead to false positive results. To address this problem, more specific real-time detection methods use labeled primers for incorporation into the amplicon, e.g. Sunrise primers [2] or Scorpion primers [3], or add additional probes to the reactions that hybridize to the amplicon in a sequence-dependent manner [4], [5]. Such DNA probes commonly carry a fluorescent label and a quencher for background control, e.g. TaqMan probes [6], and Molecular Beacons [7], [8], or two separate oligonucleotides have been used like in the case of HybProbes [9] where one oligonucleotide carries a donor dye and the other oligonucleotide carries an acceptor dye. Both HybProbes must hybridize close to each other on the PCR template to allow for detection by fluorescence resonance energy transfer (FRET). In non-hybridized TaqMan probes (or hydrolysis probes) the signal of a fluorescent dye is suppressed by a quencher, which leads to a low background signal as long as the fluorescent dye and quencher are in close contact. TaqMan probes hybridize to the template during the primer elongation step. Upon reaching the TaqMan probe, the exonuclease activity of the *Taq* DNA polymerase will start to digest the probe, which leads to the release of the fluorescent dye at the 5′ end of the probe. During the PCR cycles, the released fluorescent dye will accumulate and thus indicate the progress of the PCR at the expense of the probe. The structure of Molecular Beacons is very similar to TaqMan probes, but unbound Molecular Beacons form an internal stem loop structure that links the fluorescent label and quencher for strong background suppression. The signal of Molecular Beacons depends on a conformation change, where the fluorescent label and quencher at the ends of the probe are separated upon hybridization of the probe to the PCR template. Molecular Beacons are not digested during PCR, and therefore allow for melting curve analysis after the PCR amplification has been completed. Many other approaches to real-time PCR and fluorescent probes have been described in the literature [4], [5], [10], [11] following similar principles as outlined above for the most commonly used tools.

The use of hybridization probes is preferable for the design of highly specific PCR reactions as these probes can distinguish between different reaction products obtained using the same primer set [12] or allow for multiplex PCR where different PCR products are detected using different dyes [13]. However, each of these hybridization probes has specific features that may complicate its design such as the requirement for hairpin structures (Molecular Beacons) or do not allow post amplification analysis in dissociation studies (TaqMan probes are digested during PCR). For specific detection of mutations in genomic DNA, a combined amplification and melting curve analysis is preferable to distinguish more clearly between wild-type and mutated DNA. For melting curve analysis, it is better to run asymmetric PCR reactions, where one primer is used at a higher concentration than the other primer to enrich for the strand used as a template during melting curve analysis [14]. Multiplex assays have been developed combining different fluorescent probes and melting curve analysis [12], demonstrating the great potential of this approach for the design of new “superplexed” tests that are not limited by the availability of different dyes.

Exciton-Controlled Hybridization-sensitive Oligonucleotides (ECHO) are fluorescence labeled oligonucleotides that commonly possess a modified thymine carrying two dye moieties [15], [16], [17], [18], [19]. Using different fluorescent dyes, ECHO (also denoted as “Exciton Primers” [20] or traded as “Eprimers”) have been used in multiplexed amplification reactions by the isothermal SmartAmp2 process [20], [21], fluorescence *in situ* hybridization (ECHO-FISH) assays [22], or RNA detection in living cells [23]. ECHOs are unique for their strong background suppression involving just a single modified nucleotide, which allows for an easier synthesis and design of the oligonucleotides. Only upon hybridization to a complementary DNA or RNA strand do ECHOs generate a strong fluorescence signal caused by disrupting the excitonic interaction between the dyes and intercalation into the double-strand. Different dyes may have distinct interactions with the target DNA where base stacking and miner groove binding by cationic moieties can contribute to the binding. Depending on the dye moiety, ECHOs can show higher melting temperatures (T_M_) than standard DNA oligonucleotides of same sequence. This has been studied in detail for thiazole orange, the most commonly used dye in ECHOs [24]. Therefore short thiazole orange-labeled ECHOs can be used in melting curve analysis where they offer a wider spread of temperature differences between oligonucleotides that differ by only one base pair change. Additional dyes have been successfully used in ECHO applications [16] demonstrating the multiplex ability of ECHOs [16].

To use ECHOs as hybridization probes in real-time PCR and melting curve analysis, we prepared a modified version of ECHOs, so-called Eprobes, that are blocked at the 3′ end to prevent primer extension. Eprobes do not interfere with amplification reactions, but act as independent hybridization probes in signal detection and amplicon analysis.

## Materials and Methods

DNA oligonucleotides were purchased from Sigma-Aldrich Japan (Ishikari, Japan) and stored as 100 µM stock solutions in 10 mM Tris-HCl, 1 mM EDTA (pH 8.0). Wild-type human genomic DNA was purchased from Promega Japan (Tokyo, Japan); heterozygote human genomic DNA with the mutation L858R in exon 21 of human EGFR gene was obtained from Horizon Diagnostics (Cambridge, United Kingdom). Plasmids holding wild-type and mutated regions of the human EGFR gene were taken from [25], and plasmid DNA was prepared using a QIAprep Spin Miniprep Kit, (QIAGEN K.K., Tokyo, Japan). All DNA preparations were sequenced using a BigDye Terminator v3.1 kit (Applied Biosystems, Carlsbad, USA) and an ABI PRISM 3100-Avant capillary sequencer (Applied Biosystems, Carlsbad, USA) prior to use in PCR to confirm their correct sequences and presence of the mutation. SYBR Green I was purchased from Cambrex Bio Science Rockland Inc. (East Rutherford, USA) as a stock solution of unknown concentration. The stock solution had been diluted by 1∶300 in water for use in PCR. An EGFR gene specific TaqMan Probe (5′-FAM-TCACAGATTTTGGGCTGGCCAAAC-TAMRA-3′) was obtained from Sigma-Aldrich Japan (Ishikari, Japan) and stored in 1×TE Buffer (Promega Japan, Tokyo, Japan).

### Synthesis of Eprobes

Eprobe chemistry is derived from protocols for ECHO/Eprimer synthesis. Eprimers with paired thiazole orange moieties (D514, or in Eprobe name indicated as “TO”) are synthesized according to a modified version (T. Hanami et al. in preparation) of the previously published protocol [16], [26] on a H-8 DNA Synthesizer (Nihon Technoservice, Ushiku, Ibaraki, Japan) using phosphoramidites from Glen Research (Sterling, Virginia, USA), and 1 µmol, pore size 500 Å, Controlled Pore Glass (CPG) beads from GeneACT (Fukuoka, Japan). For the synthesis of Eprobes with paired thiazole orange or thiazole pink moieties (D570, or in Eprobe name indicated as “TP”), the Eprimer protocol was changed by replacing the standard CPG beads to 1 µmol, pore size 1000 Å, C3-Spacer-CPG from Biosearch Technologies (Novato, Californian, USA) to introduce a three carbon (C3) spacer to the 3′ end of the oligonucleotide. All Eprobes were purified on HPLC before use as described in the literature [16], [26]. To test the Eprobe chemistry, we first synthesized an Eprobe of the following sequence 5′-GAGTGCCTZGACGATAC-C3-3′, where “Z” indicates the position of the modified T (calculated m/z  = 6277.0), and confirmed its correct molecular mass (determined m/z  = 6277.8) by using a microflex instrument (Bruker Daltonics, Billerica, MA, USA) at the Support Unit for Bio-material Analysis of the RIKEN BSI Research Resources Center (Wako, Japan). Some Eprobes have also been obtained from K.K. DNAFORM (Yokohama, Japan). Refer to Table 1 for the sequences of the Eprobes prepared for this project.

**Table 1:** Eprobes used for this study.

Eprobe Name	Length(bases)	Dye(nm)	T_M_ (°C)(full match)	T_M_ (°C)(mismatch)	Sequence
Eprobe 203-10 wt TO	10	D514	52.5	39.7	TT**Z**GGGCTGG
DNA/DNA	10	–	36.6	19.4	TTTGGGCTGG
Eprobe 205-13 wt TO	13	D514	63.3	54.0	TTT**Z**GGGCTGGCC
DNA/DNA	13	–	51.7	40.3	TTTTGGGCTGGCC
Eprobe205-13m TO	13	D514	67.0	56.7	TTT**Z**GGGCGGGCC
DNA/DNA	13	–	55.8	43.4	TTTTGGGCGGGCC
Eprobe 215-21 wt TO	21	D514	77.2	72.5	TTGGGCTGGCCAAAC**Z**GCTGG
DNA/DNA	21	–	68.9	63.3	TTGGGCTGGCCAAACTGCTGG
Eprobe 215-21 wt TP	21	D570	65.2*	57.2*	TTGGGCTGGCCAAAC**Z**GCTGG
Eprobe 215-21m TO	21	D514	79.5	74.0	TTGGGCGGGCCAAAC**Z**GCTGG
DNA/DNA	21	–	71.3	65.0	TTGGGCGGGCCAAACTGCTGG
Eprobe KWT14m3 TO	14		64.8	–	AGCTGGTGGCG**Z**AG
DNA/DNA	14	D514	54.4	–	AGCTGGTGGCGTAG

(T_M_ values for TO-labeled Eprobes were estimated using the following settings: 0.05 M Na^+^, 0.002 M Mg^2+^, 0.2 µM Eprobe, 60°C Temperature; *T_M_ values for Eprobe 215-21 wt TP are actual values; in Eprobe name: TO = D514, TP = D570; Z indicating position of modified T).

### T_M_ values of Eprobes

The T_M_ values for all Eprobes labeled by D514 were calculated using an online tool described by Kimura et al. [24] under the following settings: 0.05 M Na^+^, 0.002 M Mg^2+^, 0.2 µM Eprobe, 60°C Temperature; T_M_ values for Eprobe 215-21 wt TP are actual values as the tool cannot predict T_M_ values for Eprobes labeled by D570. Predicted T_M_ values for all Eprobes and DNA oligonucleotides of the same sequence are given in Table 1. Depending on the reaction conditions, the exact T_M_ values may vary by up to some 3°C. These differences cannot be predicted, because the makers do not disclose the components of their proprietary reagent mixes. The tool can be freely accessed at: http://genome.gsc.riken.jp/echo/thermodynamics/.

### Eprobe Digestion

Eprobes (1 µM per reaction) with paired thiazole orange (Eprobe 215-21 wt TO, dye D514) or thiazole pink moieties (Eprobe 215-21 wt TP, dye D570) were fully digested with calf-intestine alkaline phosphatase (50 unit/mL, Wako Pure Chemical Industries, Tokyo, Japan), snake-venom phosphodiesterase (0.15 unit/mL, Funakoshi, Tokyo, Japan), and P1 nuclease (50 unit/mL, Wako Pure Chemical Industries, Tokyo, Japan) at 25°C for 3 h in 100 µl reaction volume. The reaction mixtures with and without digestion were measured on a RF5300PC fluorescence spectrophotometer (Shimadzu, Kyoto, Japan) with excitation at 483 nm for D514 when recording a spectrum from 495 nm to 800 nm, and an excitation at 540 nm for D570 when recording a spectrum from 550 nm to 800 nm.

### Real-time PCR

Specific primers for exon 21 in the human EGFR gene (forward primer: 5′- CCTCACAGCAGGGTCTTCTC-3′, reverse primer: 5′-CCTGGTGTCAGGAAAATGCT-3′) were taken from [27] and they amplified a 229 bp DNA fragment. Human KRAS specific primers for exons 12 and 13 (forward primer: 5′-TTATAAGGCCTGCTGAAAATGACTGAA-3′, reverse primer: 5′-TGAATTAGCTGTATCGTCAAGGCACT-3′) were taken from [28] and amplified a 92 bp DNA fragment.

Different PCR reagents and enzyme master mixes were tested for use with Eprobes during PCR. We selected AmpliTaq Gold PCR Master Mix (Applied Biosystems, Carlsbad, USA) as a *Taq* DNA polymerase with an exonuclease activity (exo+) and LightCycler 480 Genotyping Master (Roche Diagnostics, Mannheim, Germany) as a *Taq* DNA polymerase having an N-terminal deletion lacking an exonuclease activity (exo−). Suitable Eprobes concentrations were tested in the range of 25 nM to 500 nM, where 200 nM was the optimal concentration for standard reactions. The template concentration per reaction varied from 1 ng to 1000 ng genomic DNA or from 150 to 150,000,000 copies for plasmid DNA.

Amplification reactions using AmpliTaq Gold were setup in 96-well plates using 5 µl template DNA, 12.5 µl of AmpliTaq Gold PCR Master Mix (with or without 1.0 µl of GC enhancer), 0.2 µM of each primer, and 0.2 µM Eprobe in a total volume of 25 µl. Real-time PCR experiments were run on a LightCycler 480 (Roche Diagnostics, Mannheim, Germany) after activation of the hotstart enzyme for 10 min at 95°C, followed by 50 cycles of 15 s at 95°C, 30 s at 56°C, and 30 s at 72°C. Amplification signals were detected during the annealing step of each cycle at 56°C, using a SYBR Green I (483 nm–533 nm) filter for D514 and Red 610 (558–610 nm) filter for D570. For melting curve analysis, the PCR was followed by heating the reaction mixture to 95°C for 15 s, cooling to 37°C, holding at 37°C for 7 min, and then slowly heating again to 95°C at a ramp rate 2.2°C/s and continuous fluorescence acquisition at the indicated wave lengths. All PCR reactions and melting curve experiments were always performed in triplicate, and each experiment included a negative control where 1×TE Buffer (Promega Japan, Tokyo, Japan) was added instead of template DNA.

Alternatively, PCR reactions were setup using 4 µl of 5×LightCycler 480 Genotyping Master (with or without 5% Formamide), 5 µl template DNA, 0.2 µM of each primer, and 0.2 µM Eprobe in a total volume of 25 µl. Amplification reactions and melting curve analysis were performed as described above for the AmpliTaq Gold.

PCR control experiments used SYBR Green I (1/30,000 final concentration of the stock solution in PCR) or the EGFR specific TaqMan probe (50 nM) instead of Eprobes, and were run under the same reaction conditions as described above for use of AmpliTaq Gold.

To achieve a higher sensitivity in the mutation detection assays, asymmetric amplification reactions were setup using 5 µl template DNA, 12.5 µl of AmpliTaq Gold PCR Master Mix (without GC enhancer), forward to reverse primers at a 1∶3 ratio and a final concentration of 1 µM, and 0.2 µM Eprobe in a total volume of 25 µl. Real-time PCR and melting curve experiments were run as described above for use of AmpliTaq Gold.

Asymmetric amplification reactions were also performed for multiplexing detection of the human EGFR and KRAS genes using commercial genomic DNA. We mixed 500 nM of forward EGFR primer, 1500 nM of reverse EGFR primer, 250 nM of forward KRAS primer, 750 nM reverse KRAS primer, and 200 nM each of sequence specific Eprobe 215-21 wt TP for EGFR and Eprobe KWT14m3 TO for KRAS. Real-time PCR and melting curve analysis were performed as described above using AmpliTaq Gold.

### Analysis of PCR Products

PCR products were analyzed on a 3% agarose gel in 1×TBE buffer. We mixed 10 µl of each PCR reaction with 2 µl of 6×loading dye (Fermentas, Thermo Fisher Scientific K.K., Chiba, Japan) and loaded the gels together with a GeneRuler Low Range DNA Ladder, Ready-to-Use 25 to 700 bp (Fermentas, Thermo Fisher Scientific K.K., Chiba, Japan). The electrophoresis was carried out for 1 h at 100 V, and gel images were taken by a Gel Doc™ XR+ System (Bio-Rad, Hercules, CA, USA) after staining with Ethidium bromide. Images were analyzed using Image Lab 3.0 software (Bio-Rad, Hercules, CA, USA).

### PCR Data Analysis

PCR standard curves were generated by analyzing Ct values acquired from the LightCycler 480 instrument with LightCycler 480 software release 1.2.0.0625, version 1.2.0.169. The data were transferred to Microsoft Excel, and Ct values were plotted against the template DNA concentrations. Logarithmic trendlines were added, and equations and R-squared values given by Microsoft Excel are shown in the graphs. Microsoft Excel provides slopes as log_10_(x) values which were transformed into ln(x) by multiplying the slope value by 2.303 [ln(x)  = 2.303 × log_10_(x)]. The slope values were used to calculate PCR efficiencies by a Thermo Fisher online web tool at: http://www.thermoscientificbio.com/webtools/qpcrefficiency/. PCR efficiencies are based on seven different DNA concentrations and one negative control using TE buffer instead of template DNA. Amplification curves, melting curves, and derivative melting curves were obtained from the LightCycler 480 instrument as indicated above, and the data points were transferred to Microsoft Excel for plotting.

## Results

**Figure 1:**
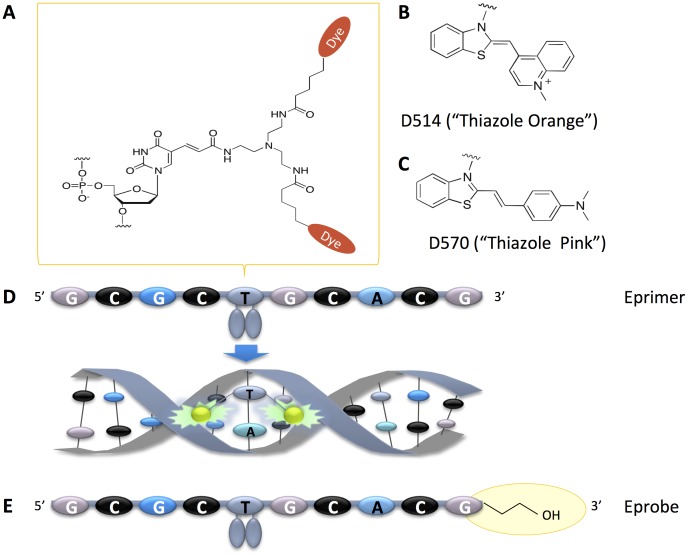
Structure and function of Eprimer and Eprobe. A. Chemical structure of modified deoxythymidine carrying two dye moieties. B. Chemical structure of D514 “Thiazole Orange”. C. Chemical structure of D570 “Thiazole Pink”. D. Signal generation by an arbitrary Eprimer (ECHO), where the two dye moieties are paired in the single-stranded oligonucleotide for signal suppression. Upon hybridization to complementary strand, dye moieties get separated and intercalate into the double-strand leading to emission of strong fluorescence. E. Structure of an arbitrary Eprobe having a blocked 3′ end to prevent primer extension during PCR.

ECHOs can easily be synthesized on a standard oligonucleotide synthesizer, where presently one deoxythymidine within the sequence is modified by an adapter to allow for the labeling with two dye moieties (Figure 1A). For Eprimers (“Exciton Primers”) used in isothermal reactions, we have further established conditions for using dyes D514 (“Thiazole Orange”, Figure 1B) and D570 (“Thiazole Pink”, Figure 1C) on real-time PCR machines [20]. Both dyes show signal suppression when incorporated into single-stranded DNA, and yield strong fluorescence upon intercalation into double-stranded DNA (Figure 1D). Eprimers carrying any of the two dyes can be used in primer extension reactions without any interference with any of the DNA polymerases we have tested so far. For use as a hybridization probe, however, it is preferable to block the 3′ end in Eprimers to avoid primer extension. We have initially tested 3′ end phosphorylation as a protection group, but this modification did not lead to stable oligonucleotides (data not shown). Therefore we selected a C3-spacer as a better 3′ end protection group that allowed for the synthesis of stable hybridization probes (Figure 1E). In line with name “Eprimer”, we call those new probes “Eprobes”.

**Figure 2:**
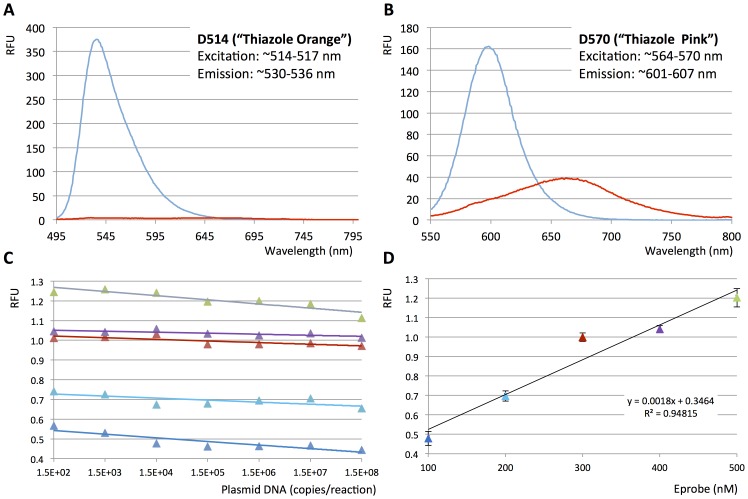
Eprobe derived background. A. Eprobe 215-21 wt TO labeled with D514 was digested as described in the Materials and Methods and fluorescence excitation spectra from 495 nm to 800 nm were recorded before (blue) and after Eprobe digestion (red). B. Eprobe 215-21 wt TP labeled with dye D570 was digested as described in the Materials and Methods and fluorescence excitation spectra from 550 nm to 800 nm were recorded before (blue) and after Eprobe digestion (red). C. Plotting random fluorescent units (RFU, mean values from triplicate data for each experiment) obtained from LightCycler 480 and Eprobe 205-13 wt TO at the beginning of the PCR reactions against plasmid DNA template concentration (from 150 to 150,000,000 copies per reaction). Eprobe concentrations are indicated by the different colors (dark blue: 100 nM, light blue: 200 nM, red: 300 nM, purple: 400 nM, light green: 500 nM). D. Plotting random fluorescent units (RFU) obtained from LightCycler 480 and Eprobe 205-13 wt TO at the beginning of the PCR reactions against Eprobe concentration (dark blue: 100 nM, light blue: 200 nM, red: 300 nM, purple: 400 nM, light green: 500 nM). For each data point, mean values plus error bars from triplicate values for each experiment and for all template concentrations are given in the graph.

Despite the strong signal suppression by the excitonic interaction between the dye moieties, unbound Eprimers and Eprobes show a weak signal in fluorescence excitation spectra as shown in Figure 2A for Eprobe 215-21 wt TO labeled with dye D514 and in Figure 2B for Eprobe 215-21 wt TP labeled with dye D570. These weak signals are nearly entirely lost upon complete digestions of the Eprobes (Figure 2A and B), indicating that destruction of an Eprobe in a reaction mix will not lead to any false positive signals. The background signal of Eprobes is most likely caused by weak intra molecular interactions between the dyes and neighboring nucleotides. Mixing Eprobes with different amounts of template DNA prior to hybridization did not lead to an increase in the background signal (Figure 2C). The total background signal strength correlates directly with the Eprobe concentration in the reaction mix (Figure 1D). We have tested different Eprobe concentrations in the range of 25 to 500 nM in PCR experiments, where a final concentration at 200 nM Eprobe per reaction offered a good signal to noise range and high sensitivity (data not shown).

**Figure 3:**
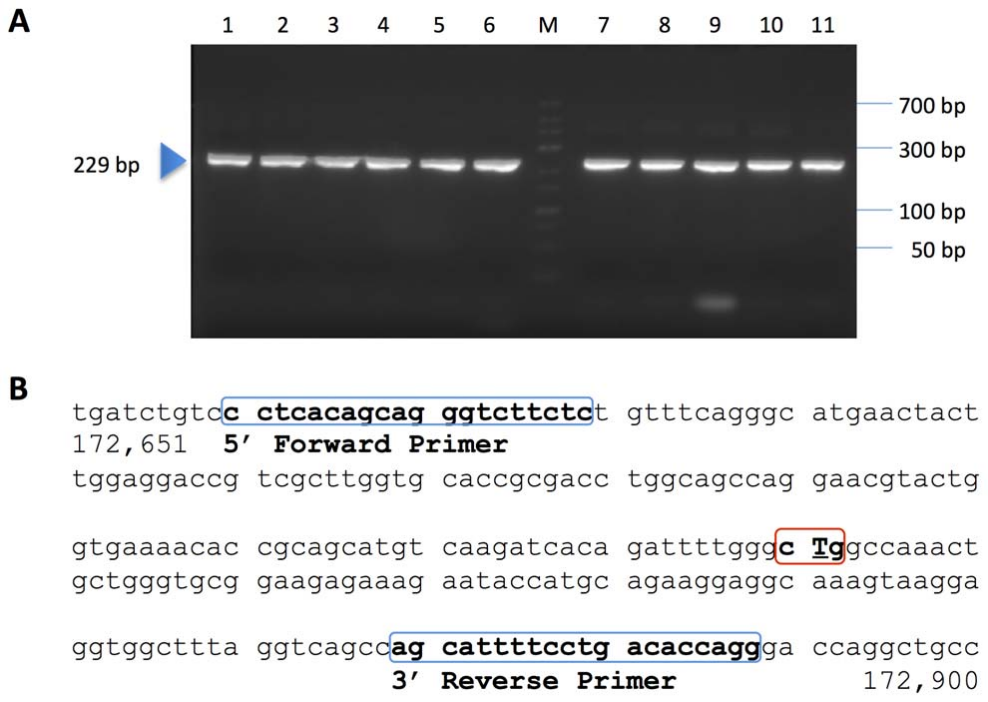
Agarose gel electrophoresis of PCR products. A: PCR products from real-time PCR experiments were analyzed on a 3% agarose gel. Lanes: 1: TaqMan Probe, 2:, Eprobe 203-10 wt TO 3: C3-blocked 203-10 wt oligonucleotide, 4: Eprobe 205-13 wt TO, 5: Eprobe205-13m TO, 6: C3-blocked 205-13 wt oligonucleotide, 7: Eprobe 215-21 wt TO, 8: Eprobe 215-21m TO, 9: C3-blocked 215-21 wt oligonucleotide, 10: Eprobe 215-21 wt TP, 11: SYBR Green I, M: marker. B: Partial sequence of human EGFR gene. Location of the primers (blue boxes) and the mutation L858R (red box) are indicated.

**Figure 4:**
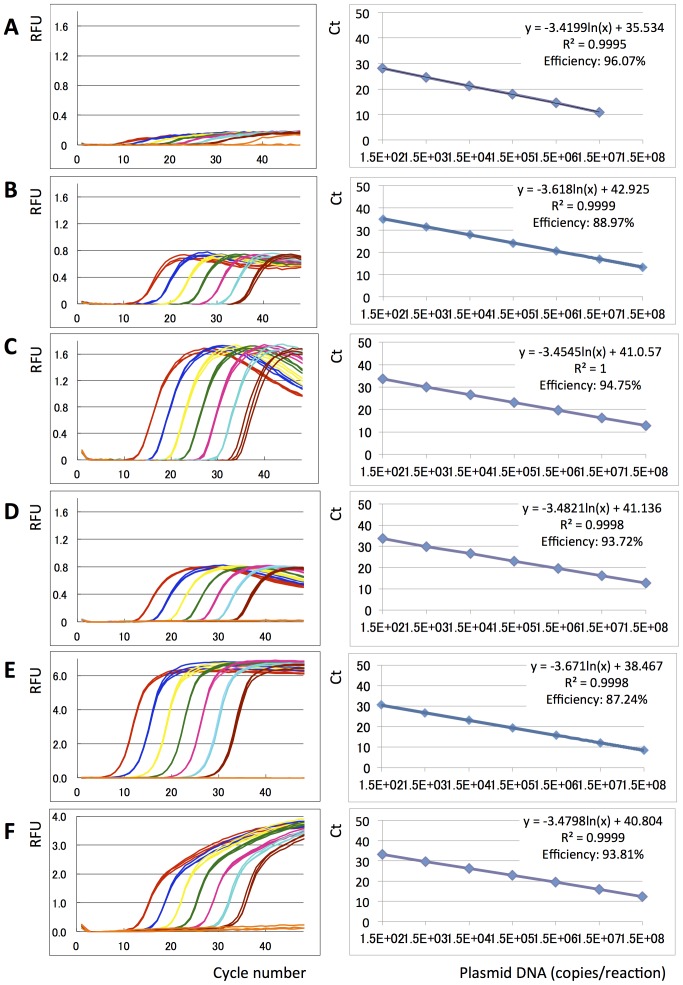
Eprobe mediated real-time PCR monitoring. Real-time PCR experiments were performed using different Eprobes, a TaqMan probe, SYBR Green I, Amplitaq and different concentrations of an EGFR wild-type plasmid DNA template. Amplification curves (Random fluorescent units (RFU) plotted against PCR cycle number) using a 7 times serial dilution of the DNA template are shown on the left, and PCR efficiency plots (Ct values plotted against logarithm of plasmid DNA concentrations) are shown on the right. Plasmid DNA concentrations are indicated by colors: Red: 1.5×10^8^ copies, Dark blue: 1.5×10^7^ copies, Yellow: 1.5×10^6^ copies, Green: 1.5×10^5^ copies, Pink: 1.5×10^4^ copies, Sky blue: 1.5×10^3^ copies, Brown: 150 copies, Orange: TE negative control. A: Eprobe 203-10 wt TO, B: Eprobe 205-13 wt TO, C: Eprobe 215-21 wt TO, D: Eprobe 215-21 wt TP, E: SYBR Green I, F: TaqMan probe. Triplicate data are shown for each experiment. For Eprobe 203-10 wt early Ct values for the highest template concentration were not recorded by the PCR machine.

The new Eprobe concept was studied for use as a hybridization probe in real-time PCR and melting curve analysis. We tested Eprobe-mediated real-time PCR conditions using exon 21 of the human epidermal growth factor receptor (EGFR) as a template [27]. This region of EGFR had been selected because of the mutation L858R (2573 T>G, COSMIC ID 6224 [29]) which is frequently found in tumors (Figure 3B). Tumor samples are often screened for the presence of this mutation considering its interference with the efficiency of anti EGFR cancer drugs [30], [31]. Eprobe-mediated detection was compared to SYBR Green I as an example for an intercalating dye and a TaqMan probe as an example for a hydrolysis probe using the same primer set and PCR conditions. In contrast to TaqMan probes, Eprobes should not be digested during PCR to keep the intact probe available for melting curve analysis after completion of the PCR. Therefore an optimal Eprobe should have a T_M_ value below the elongation temperature. The Eprobe should bind to the template DNA during the annealing step, when the fluorescent signal is detected, and would dissociate from the template again before the elongation step not to infer with the DNA polymerase. To test those considerations, we synthesized a set of specific Eprobes for detection of exon 21 in the human EGFR gene having a temperature range below and above the elongation temperature of the *Taq* DNA polymerase (Table 1). The theoretical T_M_ values for those Eprobes were estimated during the design [24] and ranked from 52.5°C (10 mer) to 77.2°C for a 21 mer (the actual T_M_ values observed during melting curve analysis varied depending on the PCR master mix used). Table 1 lists the estimated T_M_ values for all Eprobes used in this study, where the T_M_ values of Eprobes labeled by dye D514 are higher than the T_M_ values estimated for DNA oligonucleotides of the same sequence. All PCR reactions in the presence of an Eprobe yielded DNA fragments of the correct 229 bp length (Figure 3A), indicating that the 3′ end blocking group in the Eprobes indeed abolished primer extension. The blocking effect of the C3 group was further confirmed by adding unlabeled oligonucleotides of the same sequence to PCR reactions, where the C3 group also prevented primer extension (Figure 3A). Moreover, all Eprobes allowed for real-time PCR monitoring (Figure 4 A to D) and provided similar Ct values comparable to a TaqMan probe (Figure 4F); all probe derived Ct values were higher than the Ct values observed with SYBR Green I (Figure 4E). While all Eprobes showed a good linear range of detection from 150 to 150 million copies of template DNA and high PCR efficiency, the signals of the different Eprobes varied. The shortest Eprobe (203-10 wt TO) with a T_M_ below the annealing temperature (56°C) provided the weakest signals of all tested Eprobes suggesting an incomplete binding of the Eprobe at the higher annealing temperature (Figure 4A). Since our Eprobes had similar sequences, in this case hybridization efficiency may correlate with Eprobe length as the signal strength increased with Eprobe length for all D514-labeled Eprobes (Figure 4A to C). However, there had also been a trend that longer Eprobes may cause lower PCR efficiency by interfering with the elongation reaction. This effect had been observed for standard DNA oligonucleotides when using a *Taq* DNA polymerase lacking an exonuclease activity in PCR [32]. For Eprobes having the same sequence but labeled with dye D514 (Figure 4C) or D570 (Figure 4D), dye D514 provided much stronger signals in line with our previous observations from the use of both dyes in isothermal amplification reactions (data not shown).

**Figure 5:**
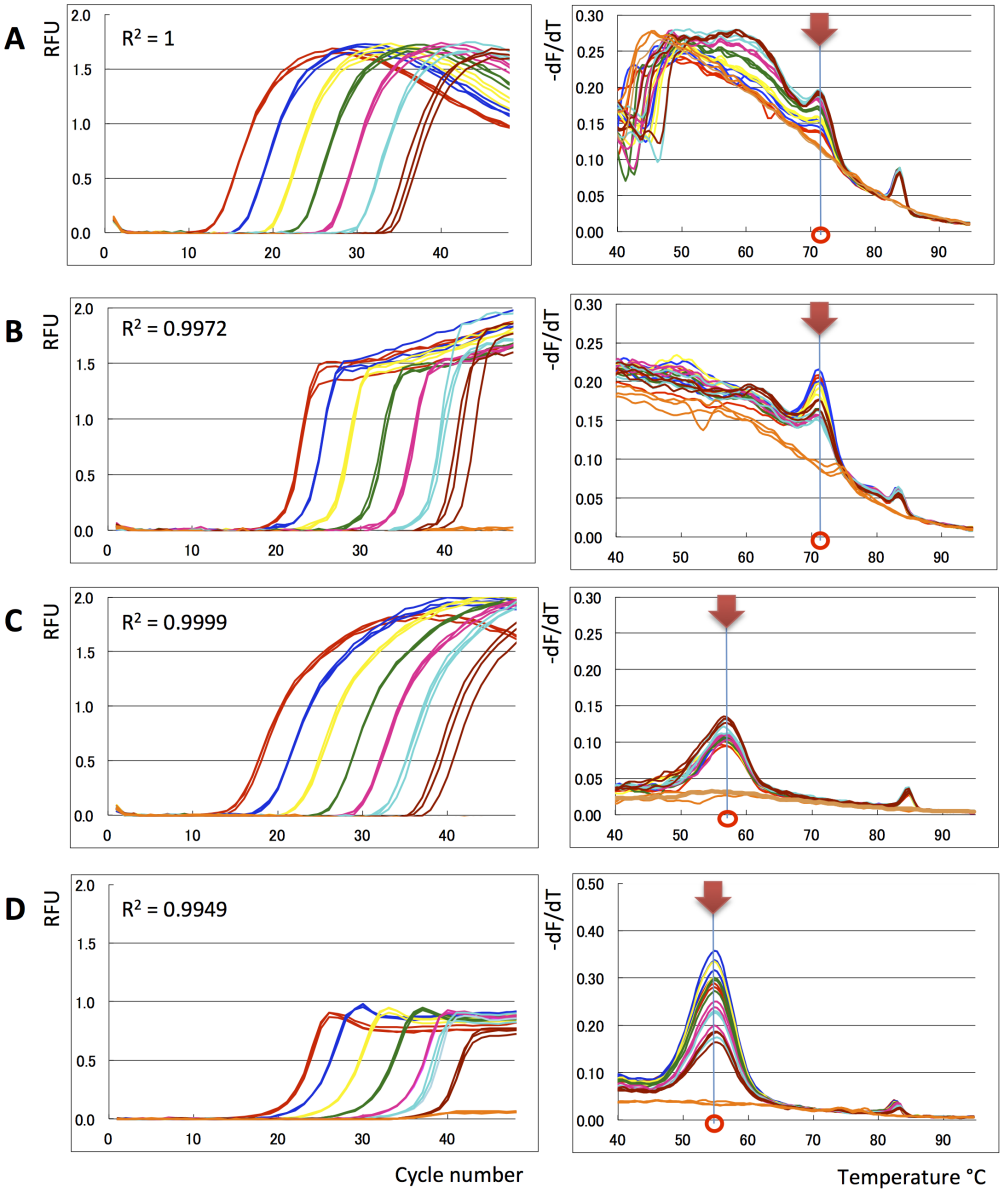
Use of exo+ and exo− *Taq* polymerase. Eprobe mediated real-time PCR experiments were performed by using an exo+ (Amplitaq) and exo− (Genotyping Master) *Taq* polymerase and Eprobes with different melting temperatures. Amplification curves (Random fluorescent units (RFU) plotted against PCR cycle number) using a 7 times serial dilution of the DNA template are shown on the left. The R-squared values of the PCR efficiency plots are indicated in the graphs. Differential melting curve analysis by plotting –dF/dT against temperature is shown on the right. Main peaks are indicated in the graph to show the different T_M_ values for both Eprobes. EGFR wild-type plasmid DNA concentrations are indicated by colors: Red: 1.5×10^8^ copies, Dark blue: 1.5×10^7^ copies, Yellow: 1.5×10^6^ copies, Green: 1.5×10^5^ copies, Pink: 1.5×10^4^ copies, Sky blue: 1.5×10^3^ copies, Brown: 150 copies, Orange: TE negative control. A: Amplitaq and Eprobe 215-21 wt TO. B: Genotyping Mastermix and Eprobe 215-21 TO. C: Amplitaq and Eprobe 205-13 wt TO. D: Genotyping Master and Eprobe 205-13 wt TO.

For the longest Eprobe (215-21 wt TO) we noticed in the forgoing PCR experiments a strong hook-like shape of the amplification curves (Figure 4C). This could have been caused by probe degradation during the PCR when using a *Taq* DNA polymerase having a 5′–3′ exonuclease activity. The consecutive reduction in the Eprobe concentration would lead to reduced signals although the amount of PCR product did no longer change at the late phase of the PCR. To test this hypothesis, we compared Eprobe 215-21 wt TO along with the shorter Eprobe 205-13 wt TO using a *Taq* DNA polymerase with a 5′–3′ exonuclease activity (Applied Biosystems AmpliTaq Gold) and a *Taq* DNA polymerase lacking the 5′–3′ exonuclease activity (Roche Genotyping Master). As shown in Figure 5, both Eprobes allowed real-time PCR monitoring using any of the two enzymes. However, melting curve analysis of the PCR reactions shown in Figure 4C showed that only Eprobe 215-21 wt TO had been degraded when using a *Taq* DNA polymerase with a 5′–3′ exonuclease activity (Figure 5A). Eprobe degradation could be avoided by using a *Taq* DNA polymerase lacking the 5′–3′ exonuclease activity (Figure 5B). The difference was less pronounced when using the shorter Eprobe 205-13 wt TO, which provided similar melting curve results for both enzymes. This indicates that Eprobes having a T_M_ below the elongation temperature are stable during PCR even in the presence of a DNA polymerase having a 5′–3′ exonuclease activity.

**Figure 6:**
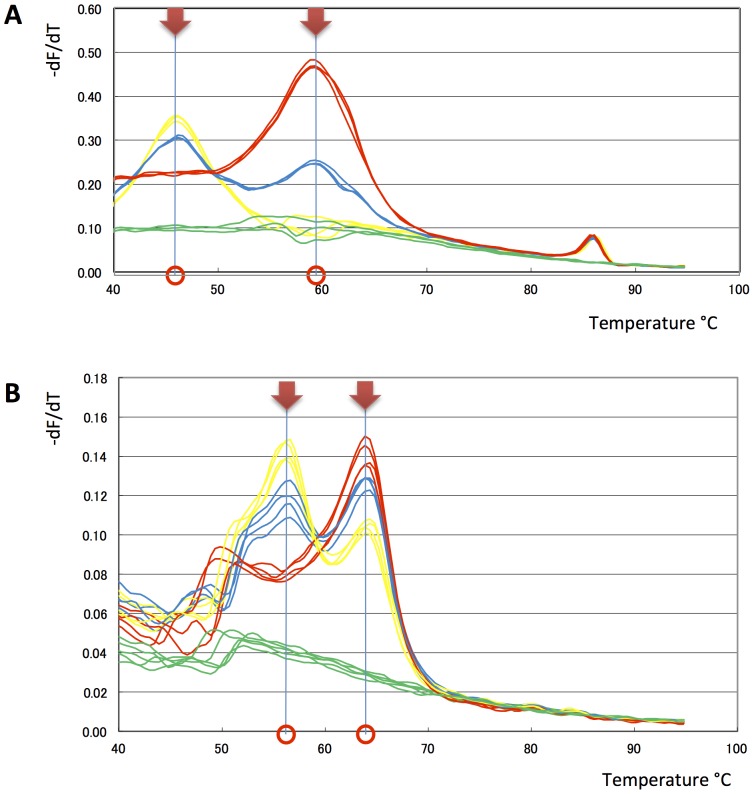
Eprobe mediated mutation detection. Asymmetric PCR was performed to enrich the reverse strand for mutation detection using different templates and Eprobes. For further details refer to Materials and Methods. A: Differential melting curve analysis by plotting –dF/dT against temperature of asymmetric PCR experiments with 5×10^4^ copies of plasmid DNA using Eprobe 205-13 wt TO. Wild-type to mutation ratios are indicated by colors (red: 100% wild-type, Yellow: 100% mutation, blue: 50% wild-type and 50% mutation, green: negative control). B: Differential melting curve analysis by plotting –dF/dT against temperature of asymmetric PCR experiments with 1 ng genomic DNA using Eprobe 215-21 wt TP. Wild-type to mutation ratios are indicated by colors (red: 100% wild-type, Yellow: 100% mutation, blue: 50% wild-type and 50% mutation, green: negative control).

Next we tested the ability of our Eprobes to distinguish between the wild-type and mutated allele of exon 21 in the human EGFR gene. In the first experiment, we mixed plasmid DNA comprising the wild-type and mutated allele at three different ratios and performed asymmetric PCR to enrich for the reverse strand in the presence of Eprobe 205-13 wt TO. The melting profiles indicated two distinct melting temperatures that are more clearly shown in the differential melting curve analysis (Figure 6A). The observed difference in the T_M_ values for the wild-type and mutated allele is in line with our estimations, although the observed T_M_ values did not match exactly with our predictions (see above). We confirmed the results of these experiments by analyzing wild-type and mutated genomic DNA with Eprobe 215-21 wt TP. Again the wild-type and mutated allele could be clearly distinguished by their different T_M_ values (Figure 6B), where the point mutation reduced the T_M_ value of the wild-type probe within the expected range. Similar results were obtained with the mutation-specific Eprobes 205-13m TO (data not shown).

**Figure 7:**
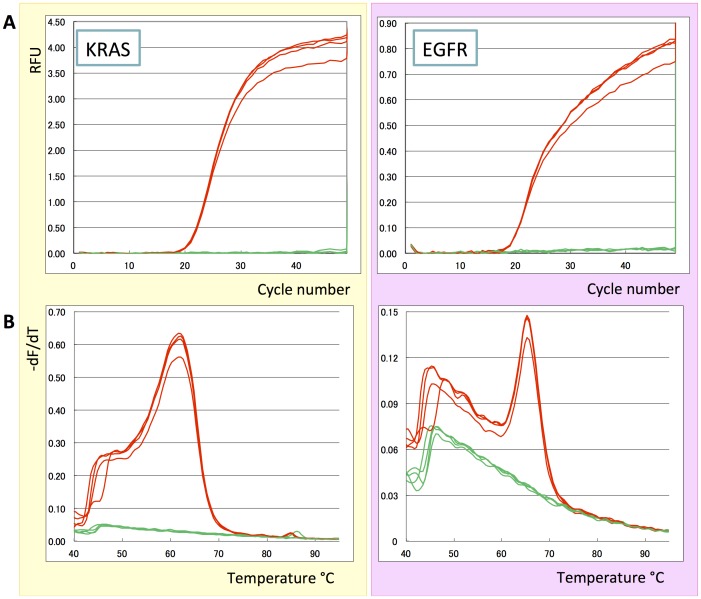
Eprobes in multiplex detection. Regions of the human EGFR and KRAS genes were amplified from genomic DNA and amplification was monitored by specific Eprobes having different dyes. A: Amplification curves plotting random fluorescent units (RFU) obtained from LightCycler 480 against PCR cycle number (Red: genomic DNA, Green: negative control). B: Differential melting curve analysis by plotting –dF/dT against temperature (Red: genomic DNA, Green: negative control). Plots in yellow on the left show signals for KRAS using dye D514 (Eprobe KWT14m3 TO); plots in pink on the right show signals for EGFR using dye D570 (Eprobe 215-21 wt TP).

In a last set of experiments, we confirmed that Eprobes having two different dyes can be used in multiplex PCR in the same way as previously shown for isothermal amplification reactions using Eprimers [20]. For this experiment, we amplified regions of the human EGFR and KRAS gene from genomic DNA with specific primer sets and Eprobes (Figure 7). Indeed the parallel amplification of both amplicons was monitored by the D570-labeled Eprobe 215-21 wt TP for the EGFR gene and the D514-labeled Eprobe KWT14m3 TO for the KRAS genes (Figure 7A). The correct synthesis of both amplicons was confirmed by melting curve analysis where the shorter Eprobe for the KRAS gene gave a lower T_M_ value (Figure 7B) as compared to the longer Eprobe used for EGFR detection. The data demonstrate that Eprobes can be used in multiplex PCR.

## Discussion

Our data demonstrate that Eprobes allow for sensitive real-time PCR monitoring. Moreover, since Eprobes under optimal conditions neither interfere nor get destroyed during the amplification reactions, they can directly be used for melting curve analysis of amplification products. Melting curve analysis is one of the most specific methods to look for mutations in DNA [33]. Even slight changes in T_M_ values can be used to distinguish single nucleotide changes in a sequence dependent manner as shown by our data for the mutation L858R in the human EGFR gene. Hence Eprobes can be applied for both quantification and qualitative analysis of amplicons on real-time PCR instruments in a single consecutive procedure combining PCR amplification and melting steps. This allows for the design of sensitive and highly specific PCR assays, for example, detection of genetically modified organisms or infectious agents [34], viral load [35], gene expression analysis [36], determination of genomic copy number variations, and allele specific discrimination of SNPs or somatic mutations.

Since Eprobes can be designed over a wide T_M_ range, their design is more flexible than for example Molecular Beacons that are also used in melting curve analysis. Molecular Beacons often have a narrow temperature range for detecting mutations. At higher temperatures, their stem loop structure melts and the high background fluorescence does not allow target detection. In contrast, the background signal of Eprobes is less affected by temperature changes. For different Eprobes we observed characteristic baseline changes with increasing temperatures (compare Figures 5 and 7). Apparently higher background signals of some Eprobes at lower temperatures related to secondary structures that break apart as the temperature increases. Therefore we commonly see a temperature-dependent decrease in the Eprobe mediated background (Y. Kimura et al. in preparation). In addition, we observed a sequence-dependence of the fluorescent signal strength. After intercalation into the double-strand, the dye moieties interact with two base pairs upstream and two base pairs downstream of the labeled nucleotide. The base pair composition in those positions determines the dye-DNA interactions, and thus affects signal strength. The signal strength of different Eprobes or Eprimers, however, has not been a limiting factor in any of our experiments. Overall, Eprobes have exceptional signal-to-noise ratios because of their exciton-controlled hybridization dependent background reduction, show a high thermal stability, and do not produce any false positive signals upon probe degradation. This is advantageous when unpurified DNA templates are used, or if an Eprobe gets partially degraded during extended storage, repeated freeze-thawing cycles, or upon exposure to light. Eprobe-mediated signals can be further increased, if needed, by using higher Eprobe concentrations than the 200 nM range we routinely used in our studies. The Eprobe mediated signals correlate directly with the Eprobe concentration (Figure 2 and data not shown) for up to a concentration of 500 nM Eprobe. Hence higher Eprobe concentrations may be used for highly sensitive real-time PCR experiments.

ECHOs labeled by dye D514 have been studied in detail for their thermodynamic properties [24]. Further, general rules for the design of ECHOs have been published in the literature [19]. Based on those data and our own ongoing studies for an optimization of Eprimers and Eprobes, we are developing online tools for easy design of Eprobe-mediated real-time PCR experiments (Y. Kimura unpublished data). In principle, Eprobe design follows a few basic rules: (i) labeling one nucleotide (presently dT and dC can be labeled in ECHOs) is sufficient for very strong signals; (ii) the modified base should be in the center of the oligonucleotide rather than at the very ends of the probe; (iii) Eprobes should not form secondary structures or hybridize to each other; (iv) Eprobe length and sequence have to be adjusted to expected melting temperatures in line with the annealing temperature and enzyme used in the PCR. Eprobes work over a wide T_M_ range allowing for variable lengths and lower sequence requirements than other hybridization probes used in PCR. Shorter Eprobes could avoid G/C or A/T rich regions, whereas longer Eprobes could more easily distinguish between problematic target sequences, e.g. containing repeat elements. Additional considerations should be given to the use of *Taq* DNA polymerases with or without a 5′–3′ exonuclease activity [37], [38] or other thermostable DNA polymerases suitable for PCR applications. While even partially degraded Eprobes still allow for real-time monitoring, they are not suitable for melting curve analysis. Accordingly, we recommend designing Eprobes with a T_M_ value of about 5°C below elongation temperature or lower when using a DNA polymerase having a 5′–3′ exonuclease activity. Switching to DNA polymerases lacking any 5′–3′ exonuclease activity offers a wider temperature range for designing Eprobes, which may prove very useful for reactions using multiple Eprobes having distinct T_M_ values. Eprobes with T_M_ values below the annealing temperature are not suitable for real-time monitoring of PCR, but are still functional in melting curve analysis using a wider temperature range. Combining multiple Eprobes with different dyes and melting temperatures could become an attractive approach for the design of highly complex “superplexed” PCR assays. Here we demonstrated the use of Eprobes having two different dyes in multiplex PCR. Besides dyes D514 and D570, additional dyes have been described in the literature for use in ECHOs [16]. However, we found that for example dye D640 is not thermostable and hence such a dye cannot be used in PCR. Therefore additional thermostable dyes for Eprobes are desirable to extend the multiplex abilities of Eprobes in the future.

For genotyping experiments, we performed asymmetric PCR reactions followed by melting curve analysis [14]. Asymmetric PCR performed well with all the different Eprobes tested, and we recommend its use in Eprobe mediated SNP and mutation detection experiments. Because of the higher DNA binding affinity of D514-labeled Eprobes compared to standard DNA oligonucleotides, such Eprobes are preferable for melting curve analysis, as they provide exceptionally specific and sensitive mutation detection. In contrast to D514, the second dye used in our studies (D570) does not stabilize DNA binding to the same effect as D514 (compare T_M_ values in Table 1 for Eprobes 215-21 wt TO and 215-21 wt TP, both having the same sequence). Most likely, the stabilizing effect of a dye depends on how it intercalates into the double-strand and its interactions with the neighboring nucleotides. Therefore every new dye used for Eprobes should be individually tested for its thermodynamic properties [24] and effect on DNA or RNA binding.

We recommend Eprobes rather than previously published ECHOs or Eprimers for all PCR applications involving real-time monitoring and melting curve analysis for extended template characterization. Only Eprobes can provide the additional specificity offered by working with a primer-independent hybridization probe during PCR. Moreover, Eprobes hold great potential for the development of *in situ* hybridization assays for *ex vivo* and *in vivo* detection in FISH assays making them universal hybridization probes.

## References

[1] Morrison TB, Weis JJ, Wittwer CT (1998) Quantification of low-copy transcripts by continuous SYBR Green I monitoring during amplification. Biotechniques 24: 954–958, 960, 962. 9631186

[2] Nazarenko IA, Bhatnagar SK, Hohman RJ (1997) A closed tube format for amplification and detection of DNA based on energy transfer. Nucleic Acids Res 25: 2516–2521. 9171107 10.1093/nar/25.12.2516 PMC146748

[3] Whitcombe D, Theaker J, Guy SP, Brown T, Little S (1999) Detection of PCR products using self-probing amplicons and fluorescence. Nature biotechnology 17: 804–807. 10.1038/11751 10429248

[4] VanGuilder HD, Vrana KE, Freeman WM (2008) Twenty-five years of quantitative PCR for gene expression analysis. Biotechniques 44: 619–626. 18474036 10.2144/000112776

[5] Mackay IM, Arden KE, Nitsche A (2004) Real-time Fluorescent PCR Techniques to Study Microbial–Host Interactions. Methods in Microbiology 34: 255–330. 10.1016/S0580-9517(04)34010-9 PMC7148886 38620210

[6] Lee LG, Connell CR, Bloch W (1993) Allelic discrimination by nick-translation PCR with fluorogenic probes. Nucleic Acids Res 21: 3761–3766. 8367293 10.1093/nar/21.16.3761 PMC309885

[7] Tyagi S, Kramer FR (1996) Molecular beacons: probes that fluoresce upon hybridization. Nature biotechnology 14: 303–308. 10.1038/nbt0396-303 9630890

[8] Tyagi S, Kramer FR (2012) Molecular beacons in diagnostics. F1000 Med Rep 4: 10. 22619695 10.3410/M4-10 PMC3357010

[9] Caplin BE, Rasmussen RP, Bernard PS, Wittwer CT (1999) LightCycler™ Hybridization Probes: The most direct way to monitor PCR amplification for quantification and mutation detection. Biochemica Roche Molecular Biochemicals 1: 5–8.

[10] Buh Gasparic M, Cankar K, Zel J, Gruden K (2008) Comparison of different real-time PCR chemistries and their suitability for detection and quantification of genetically modified organisms. BMC Biotechnol 8: 26. 18325084 10.1186/1472-6750-8-26 PMC2322970

[11] Juskowiak B (2011) Nucleic acid-based fluorescent probes and their analytical potential. Anal Bioanal Chem 399: 3157–3176. 21046088 10.1007/s00216-010-4304-5 PMC3044240

[12] Huang Q, Liu Z, Liao Y, Chen X, Zhang Y, et al (2011) Multiplex fluorescence melting curve analysis for mutation detection with dual-labeled, self-quenched probes. PLoS One 6: e19206. 21552536 10.1371/journal.pone.0019206 PMC3084284

[13] Mackay IM, Arden KE, Nitsche A (2002) Real-time PCR in virology. Nucleic Acids Res 30: 1292–1305. 11884626 10.1093/nar/30.6.1292 PMC101343

[14] Szilvasi A, Andrikovics H, Kalmar L, Bors A, Tordai A (2005) Asymmetric PCR increases efficiency of melting peak analysis on the LightCycler. Clin Biochem 38: 727–730. 15982647 10.1016/j.clinbiochem.2005.04.015

[15] Ikeda S, Kubota T, Yanagisawa H, Yuki M, Okamoto A (2009) Synthesis of exciton-controlled fluorescent probes for RNA imaging. Nucleic Acids Symp Ser (Oxf): 155–156. 10.1093/nass/nrp078 19749307

[16] Ikeda S, Kubota T, Yuki M, Okamoto A (2009) Exciton-controlled hybridization-sensitive fluorescent probes: multicolor detection of nucleic acids. Angew Chem Int Ed Engl 48: 6480–6484. 19637175 10.1002/anie.200902000

[17] Okamoto A (2011) ECHO probes: a concept of fluorescence control for practical nucleic acid sensing. Chem Soc Rev 40: 5815–5828. 21660343 10.1039/c1cs15025a

[18] Okamoto A, Ikeda S, Kubota T, Yuki M, Yanagisawa H (2009) Exciton-controlled fluorescence: application to hybridization-sensitive fluorescent DNA probe. Nucleic Acids Symp Ser (Oxf): 49–50. 10.1093/nass/nrp025 19749254

[19] Wang DO, Okamoto A (2012) ECHO probes: fluorescence emission control for nucleic acid imaging. J Photochem Photobiol C-Photochem Rev 13: 112–123.

[20] Lezhava A, Ishidao T, Ishizu Y, Naito K, Hanami T, et al (2010) Exciton Primer-mediated SNP detection in SmartAmp2 reactions. Hum Mutat 31: 208–217. 20052755 10.1002/humu.21177

[21] Kawai Y, Kimura Y, Lezhava A, Kanamori H, Usui K, et al (2012) One-step detection of the 2009 pandemic influenza A(H1N1) virus by the RT-SmartAmp assay and its clinical validation. PLoS One 7: e30236. 22295077 10.1371/journal.pone.0030236 PMC3266250

[22] Wang DO, Matsuno H, Ikeda S, Nakamura A, Yanagisawa H, et al (2012) A quick and simple FISH protocol with hybridization-sensitive fluorescent linear oligodeoxynucleotide probes. RNA 18: 166–175. 22101241 10.1261/rna.028431.111 PMC3261739

[23] Kubota T, Ikeda S, Yanagisawa H, Yuki M, Okamoto A (2010) Sets of RNA repeated tags and hybridization-sensitive fluorescent probes for distinct images of RNA in a living cell. PLoS One 5: e13003. 20885944 10.1371/journal.pone.0013003 PMC2946342

[24] Kimura Y, Hanami T, Tanaka Y, de Hoon MJ, Soma T, et al (2012) Effect of thiazole orange doubly labeled thymidine on DNA duplex formation. Biochemistry 51: 6056–6067. 22765348 10.1021/bi300293d

[25] Hoshi K, Takakura H, Mitani Y, Tatsumi K, Momiyama N, et al (2007) Rapid detection of epidermal growth factor receptor mutations in lung cancer by the SMart-Amplification Process. Clinical cancer research : an official journal of the American Association for Cancer Research 13: 4974–4983. 17785547 10.1158/1078-0432.CCR-07-0509

[26] Ikeda S, Okamoto A (2008) Hybridization-sensitive on-off DNA probe: application of the exciton coupling effect to effective fluorescence quenching. Chemistry, an Asian journal 3: 958–968. 10.1002/asia.200800014 18446920

[27] Weber F, Fukino K, Sawada T, Williams N, Sweet K, et al (2005) Variability in organ-specific EGFR mutational spectra in tumour epithelium and stroma may be the biological basis for differential responses to tyrosine kinase inhibitors. Br J Cancer 92: 1922–1926. 15841079 10.1038/sj.bjc.6602557 PMC2361765

[28] Krypuy M, Newnham GM, Thomas DM, Conron M, Dobrovic A (2006) High resolution melting analysis for the rapid and sensitive detection of mutations in clinical samples: KRAS codon 12 and 13 mutations in non-small cell lung cancer. BMC Cancer 6: 295. 17184525 10.1186/1471-2407-6-295 PMC1769510

[29] Forbes SA, Bhamra G, Bamford S, Dawson E, Kok C, et al. (2008) The Catalogue of Somatic Mutations in Cancer (COSMIC). Current protocols in human genetics/editorial board, Jonathan L Haines [et al] Chapter 10: Unit 10 11. 10.1002/0471142905.hg1011s57 PMC2705836 18428421

[30] Rosell R, Taron M, Reguart N, Isla D, Moran T (2006) Epidermal growth factor receptor activation: how exon 19 and 21 mutations changed our understanding of the pathway. Clinical cancer research : an official journal of the American Association for Cancer Research 12: 7222–7231. 17189393 10.1158/1078-0432.CCR-06-0627

[31] Paez JG, Janne PA, Lee JC, Tracy S, Greulich H, et al (2004) EGFR mutations in lung cancer: correlation with clinical response to gefitinib therapy. Science 304: 1497–1500. 15118125 10.1126/science.1099314

[32] Yu D, Mukai M, Liu Q, Steinman CR (1997) Specific inhibition of PCR by non-extendable oligonucleotides using a 5′ to 3′ exonuclease-deficient DNA polymerase. Biotechniques 23: 714–716, 718–720. 10.2144/97234st06 9343698

[33] Erali M, Voelkerding KV, Wittwer CT (2008) High resolution melting applications for clinical laboratory medicine. Exp Mol Pathol 85: 50–58. 18502416 10.1016/j.yexmp.2008.03.012 PMC2606052

[34] Henriques A, Carvalho F, Pombinho R, Reis O, Sousa S, et al. (2012) PCR-based screening of targeted mutants for the fast and simultaneous identification of bacterial virulence factors. Biotechniques 2012. 10.2144/000113906 26307257

[35] Ratcliff RM, Chang G, Kok T, Sloots TP (2007) Molecular diagnosis of medical viruses. Current issues in molecular biology 9: 87–102. 17489437

[36] Lu S, Smith AP, Moore D, Lee NM (2010) Different real-time PCR systems yield different gene expression values. Mol Cell Probes 24: 315–320. 20416369 10.1016/j.mcp.2010.04.002

[37] Lyamichev V, Brow MA, Varvel VE, Dahlberg JE (1999) Comparison of the 5' nuclease activities of taq DNA polymerase and its isolated nuclease domain. Proc Natl Acad Sci U S A 96: 6143–6148. 10339555 10.1073/pnas.96.11.6143 PMC26849

[38] Wilhelm J, Pingoud A, Hahn M (2001) Comparison between Taq DNA polymerase and its Stoffel fragment for quantitative real-time PCR with hybridization probes. Biotechniques 30: 1052–1056, 1058, 1060 passim. 10.2144/01305rr04 11355341

